# The fourth report of the European Registry for Patients with Mechanical Circulatory Support (EUROMACS) of the European Association for Cardiothoracic Surgery: focus on standardized outcome ratios

**DOI:** 10.1093/ejcts/ezaf016

**Published:** 2025-01-28

**Authors:** Kevin M Veen, Mazen Ahmed, Christoffer Stark, Luca Botta, Kyriakos Anastasiadis, Alexander Bernhardt, Michael Berchtold-Herz, Kadir Caliskan, David Reineke, Kevin Damman, Arnt Fiane, Angeliki Gkouziouta, Can Gollmann-Tepeköylü, Emil Najjar, Michal Hulman, Attilio Iacovoni, Antonio Loforte, Bela Merkely, Francesco Musumeci, Marina Comisso, Petr Němec, Ivan Netuka, Mustafa Özbaran, Evgenij Potapov, Yuri Pya, Gregorio Rábago, Faiz Ramjankhan, Anna Mara Scandroglio, Marina Pieri, Hermann Reichenspurner, Alexey Dashkevich, Bernard Stockman, Marc Vanderheyden, Laurens Tops, Thorsten Wahlers, Piotr Przybyłowski, Daniel Zimpfer, Brian Bridal Løgstrup, David Santer, Gloria Färber, Jan Gummert, Bart Meyns, Theo M M H de By, Felix Schoenrath

**Affiliations:** Department of Cardiothoracic Surgery, Erasmus MC, Rotterdam, The Netherlands; EUROMACS, EACTS House, Windsor, UK; Department of Cardiac Surgery, Helsinki University Hospital, Helsinki, Finland; IRCCS AOU di Bologna Sant’Orsola, Dept of cardiac surgery, San Orsola Hospital, Bologna, Italy; Cardiothoracic Department, Aristotle University, AHEPA University Hospital, Thessaloniki, Greece; Department of Cardiovascular Surgery, University Heart and Vascular Center Hamburg, Hamburg, Germany; Department of Cardiovascular Surgery, University Hospital Freiburg, Freiburg, Germany; Department of Cardiology, Thorax Center, Cardiovascular Institute, Erasmus MC University Medical Center, Rotterdam, Netherlands; Department of Cardiovascular Surgery, University Hospital Bern, Switzerland; Universitair Medisch Centrum Groningen, University of Groningen, Groningen, Netherlands; Department of Cardiothoracic Surgery, Rikshospitalet, Oslo, Norway; Onassis Cardiac Surgery Center, Athens, Greece; Department of Cardiac Surgery, Medical University of Innsbruck, Innsbruck, Austria; Department of Medicine, Karolinska University Hospital, Stockholm, Sweden; Department of Cardiac Surgery, Klinika Kardiochirurgie NUSCH, Bratislava, Slovakia; Department of Transplant Surgery and Surgical Treatment of Heart Failure, Ospedale Papa Giovanni XIII, Bergamo, Italy; Department of Cardiothoracic, Transplantation and Vascular Surgery, Azienda Ospedaliero-Universitaria Citta della Salute e della Scienza Torino, Turin, Italy; Department of Cardiology, Heart Center of the Semmelweis University; Cardiac Surgery and Heart Transplantation Unit, Azienda Ospedaliera San Camillo-Forlanini, Rome, Italy; Cardiac Surgery and Heart Transplantation Unit, Azienda Ospedaliera San Camillo-Forlanini, Rome, Italy; Department of Cardiovascular Surgery and Transplantations, Center for Cardiovascular Surgery and Transplantation Surgery, Brno, Czech Republic; Department of Cardiovascular Surgery, Institute for Clinical and Experimental Medicine (IKEM), Prague, Czech Republic; Department of Cardiovascular Surgery, Ege University Hospital, Izmir, Turkey; Department of Cardiothoracic and Vascular Surgery, Deutsches Herzzentrum der Charité (DHZC), Berlin, Germany; DZHK (German Centre for Cardiovascular Research), Berlin, Germany; National Research Cardiac Surgery Center, Astana, Kazakhstan; Cardiology and Cardiac Surgery Department, Clinica Universidad de Navarra, Pamplona, Spain; Department of Cardiothoracic Surgery, Utrecht University Medical Center, Utrecht, Netherlands; Department of Anesthesia and Intensive Care, IRCCS San Raffaele Scientific Institute, Milano, Italy; Department of Anesthesia and Intensive Care, IRCCS San Raffaele Scientific Institute, Milano, Italy; Department of Cardiovascular surgery, University Hospital Hamburg-Eppendorf, Hamburg, Germany; Department of Cardiac Surgery, Herzzentrum Leipzig, Leipzig, Germany; Department of Cardiovascular Surgery, Onze Lieve Vrouwenziekenhuis, Aalst, Belgium; Department of Cardiovascular Surgery, Onze Lieve Vrouwenziekenhuis, Aalst, Belgium; Department of Cardiology, Leiden University Medical Center, Leiden, Netherlands; Department for Cardiothoracic Surgery Heart Center, Universitätsklinikum Köln AöR, Köln, Germany; Silesian Center for Heart Diseases, Zabrze, Poland; Department of Cardiac Surgery, Vienna Medical University, Vienna, Austria; Department of Cardiology, Arhus University Hospital, Arhus, Denmark; Department of Cardiac Surgery, University Hospital Basel, Basel, Switzerland; Department of Cardiac Surgery, Saarland University Medical Center, Homburg, Germany; Universitäts-Herzzentrum Thüringen, Jena, Germany; Department of Thoracic and Cardiovascular Surgery, Herz- und Diabeteszentrum NRW, Bad Oeynhausen, Germany; Department of Cardiac Surgery, Katholieke Universiteit Leuven, Leuven, Belgium; EUROMACS, EACTS House, Windsor, UK; Department of Cardiothoracic and Vascular Surgery, Deutsches Herzzentrum der Charité (DHZC), Berlin, Germany

**Keywords:** Mechanical circulatory support, Left ventricular assist device, EUROMACS

## Abstract

**OBJECTIVES:**

This 4th report aimed to provide insights into patient characteristics, outcomes and standardized outcome ratios of patients implanted with durable Mechanical Circulatory Support across participating centres in the European Registry for Patients with Mechanical Circulatory Support (EUROMACS) registry.

**METHODS:**

All registered patients receiving durable mechanical circulatory support up to August 2024 were included. The expected number of events was predicted using penalized logistic regression. Standardized outcome ratios (Observed/Expected events) were presented in plots to assess 30-day and 1-year mortality, ischaemic stroke and major bleeding outcomes. Expected events were estimated using penalized logistic regression using demographics and comorbidities as predictors. Centres with <90% follow-up completeness were excluded from standardized outcome ratio assessment.

**RESULTS:**

Analysis included 6962 implants in 6408 patients (457 patients underwent repeated implants) registered in EUROMACS from 17 countries (32 centres) (median age: 58 years, 83% males, 17% Interagency Registry for Mechanically Assisted Circulatory Support class 1). Thirty-day mortality, major bleeding and ischaemic stroke probabilities were 9.6, 12.6% and 2.1%, respectively. Standardized mortality ratios showed variability between centres, ranging from 0 (95% CI 0–0) to 1.4 (95% CI 1.2–1.7). Higher standardized bleeding outcome ratios correlated with higher standardized ischaemic stroke ratio’s (Spearman *r*: 0.56, *P* = 0.008).

**CONCLUSIONS:**

Most included centres perform as expected given the demographics and comorbidities of patients. A positive correlation was found between standardized bleeding and ischaemic stroke ratios, reflecting the need of continuously monitoring of adverse events by quality improvement programs.

## Graphical abstract

**Figure:**
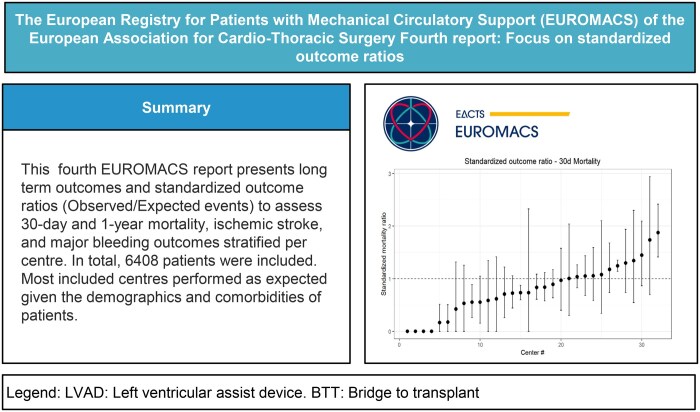

## INTRODUCTION

Durable mechanical circulatory support (MCS) has emerged as a standard for end-stage heart failure treatment, either as bridge to transplant, bridge to decision or destination therapy (DT) [1]. To ensure the monitoring of quality care for these patients, extensive databases serve as invaluable resources for both quality improvement initiatives and research endeavours. The European Registry for Patients with Mechanical Circulatory Support (EUROMACS), one of the official databases of the European Association for Cardio-Thoracic Surgery (EACTS) offers a robust repository of clinical data on long-term outcomes in patients receiving durable MCS. Since its establishment in 2011 [2], EUROMACS has been instrumental in tracking patient outcomes, serving as a benchmark for healthcare providers across Europe.

This 4th annual report details patient characteristics and outcomes, with a particular focus on standardized outcome ratios of mortality, bleeding and stroke per centre.

## METHODS

The EUROMACS registry is governed by the EACTS Council and embedded in the Quality Improvement Program (QUIP). The Council is advised by the EUROMACS Committee with respect to its strategy and policy. EUROMACS data can be requested via a controlled data access mechanism by submitting a Research Protocol to the EUROMACS Committee and Scientific Review Panel [2, 3]. Each participating centre obtained local medical ethical committee or institutional review board approval to collect and share data with the EUROMACS registry.

### Data selection

**Figure 1:**
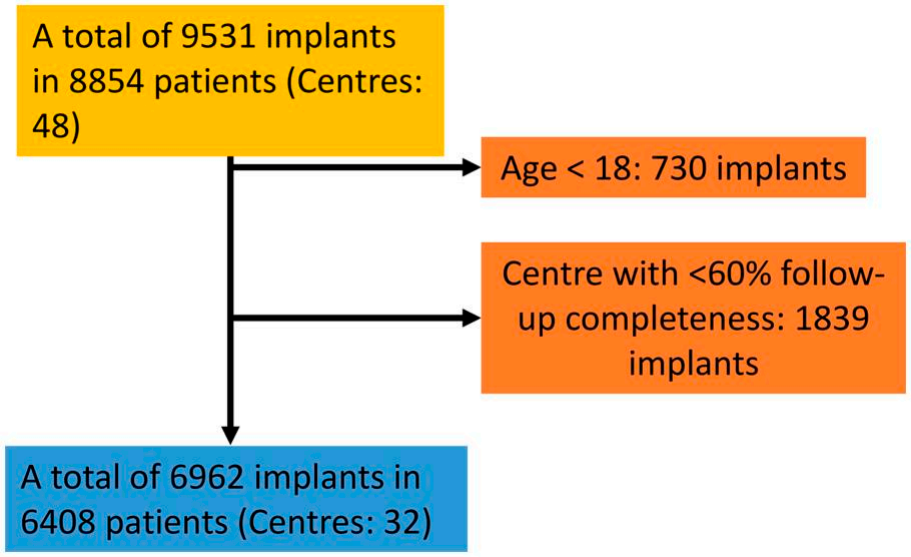
Flowchart of included patients.

Data selection and analysis were done in August 2024. Temporal analyses are shown until 31 December 2023. Procedures in children were excluded and centres with <60% follow-up completeness according to the Clark C method were excluded [4]. Follow-up completeness of excluded centres is presented in Supplementary Material, Table S1. A flow chart of included patients is shown in Fig. 1.

### Primary outcomes

The primary outcome was mortality, and secondary outcomes included major ischaemic stroke and major bleeding. Outcomes were defined according to the Interagency Registry for Mechanically Assisted Circulatory Support (INTERMACS) 2014 definitions (Supplementary Material, File 1). In this report, major bleedings also included haemorrhagic strokes.

### Standardized outcome ratios

Standardized outcome ratios per centre were calculated by dividing the cumulative observed frequency by the cumulative expected frequency. A standardized outcome ratio of 1 reflects that the centre’s outcomes are exactly as expected. A standardized outcome ratio >1 indicates the outcomes are worse than expected, whereas a standardized outcome ratio <1 indicates outcomes are better than expected.

Expected outcome frequency was estimated for 30 days, and 1-year mortality, bleeding and ischaemic stroke using a penalized logistic regression (least absolute shrinkage and selection operator). This machine learning approach performs both variable selection and regularization to enhance prediction accuracy and interpretability, and hence protects against overfitting in the setting of limited events [5]. Hyperparameters were tuned using 10-fold cross validation [6]. Variables used in the models are shown in Supplementary Material, Table S2. Model discrimination was assessed using Area under the Curve and calibration using calibration plots. Missing baseline data with <40% missing values were imputed using multiple imputation. Centres that had <90% completeness (naïve) of follow-up were excluded for these analyses. Patients censored before 30 days and 1-year follow-up were excluded. Details on the analytic approach are specified in Supplementary Material, Text 1 and Supplementary Material, Table S3.

Standardized outcome ratios were presented in plots, with the anonymized centres on the *x*-axis and standardized outcome on the *y*-axis. Bootstrapped 95% confidence intervals were generated using the percentile method, using 10 000 resamples.

### Statistical analyses

Continuous data are presented as mean (standard deviation) (Gaussian distribution) or median [interquartile range] (non-Gaussian distribution). Categorical data are presented as frequencies (percentage). Comparisons among continuous variables were made with the one-way analysis of variance or the Kruskal–Wallis test. Comparisons of categorical variables were made with the χ^2^ test or with the Fisher’s exact test. Mortality is a competing risk with explant and transplant, and the Aalen–Johannsen estimator was used to construct multi-state curves [7]. Length of follow-up was calculated as the median of observed time on device.

## RESULTS

**Figure 2:**
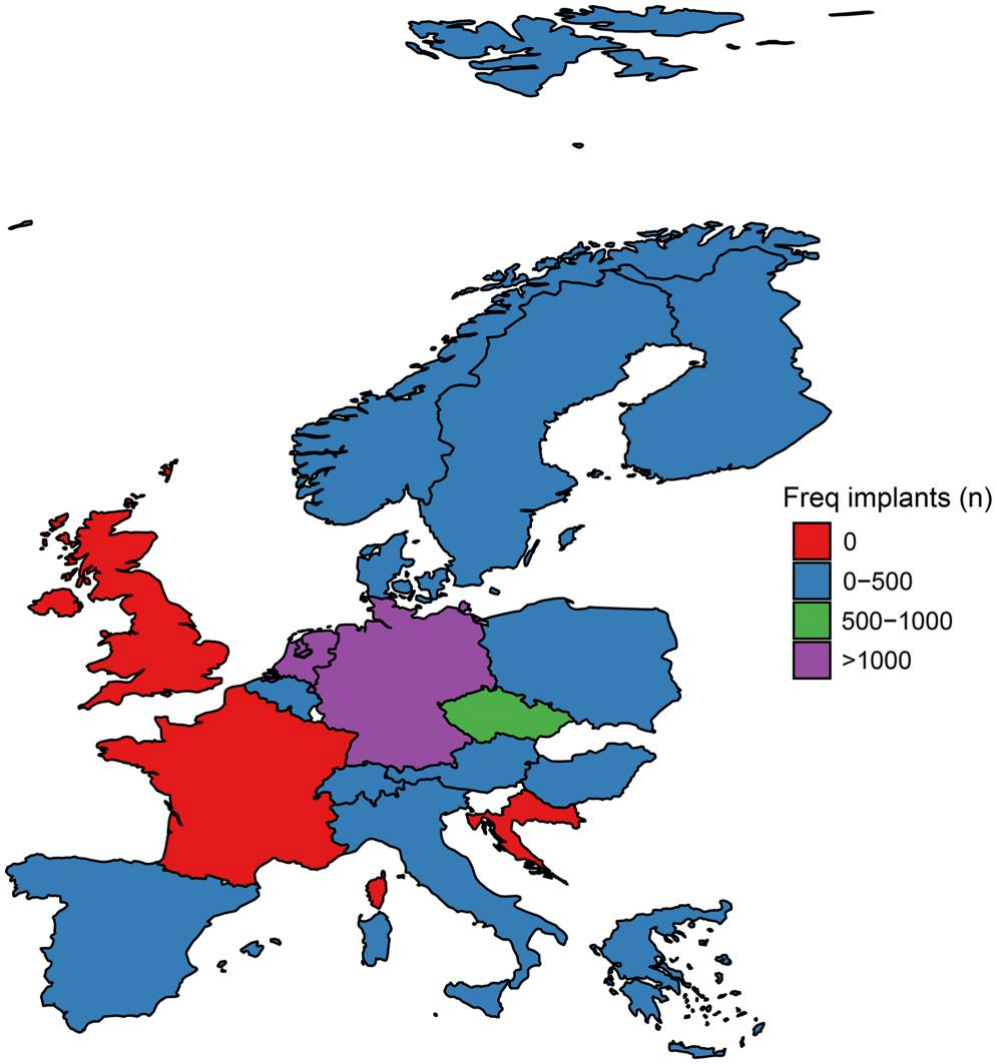
Frequency of tracked implants per country.

**Figure 3:**
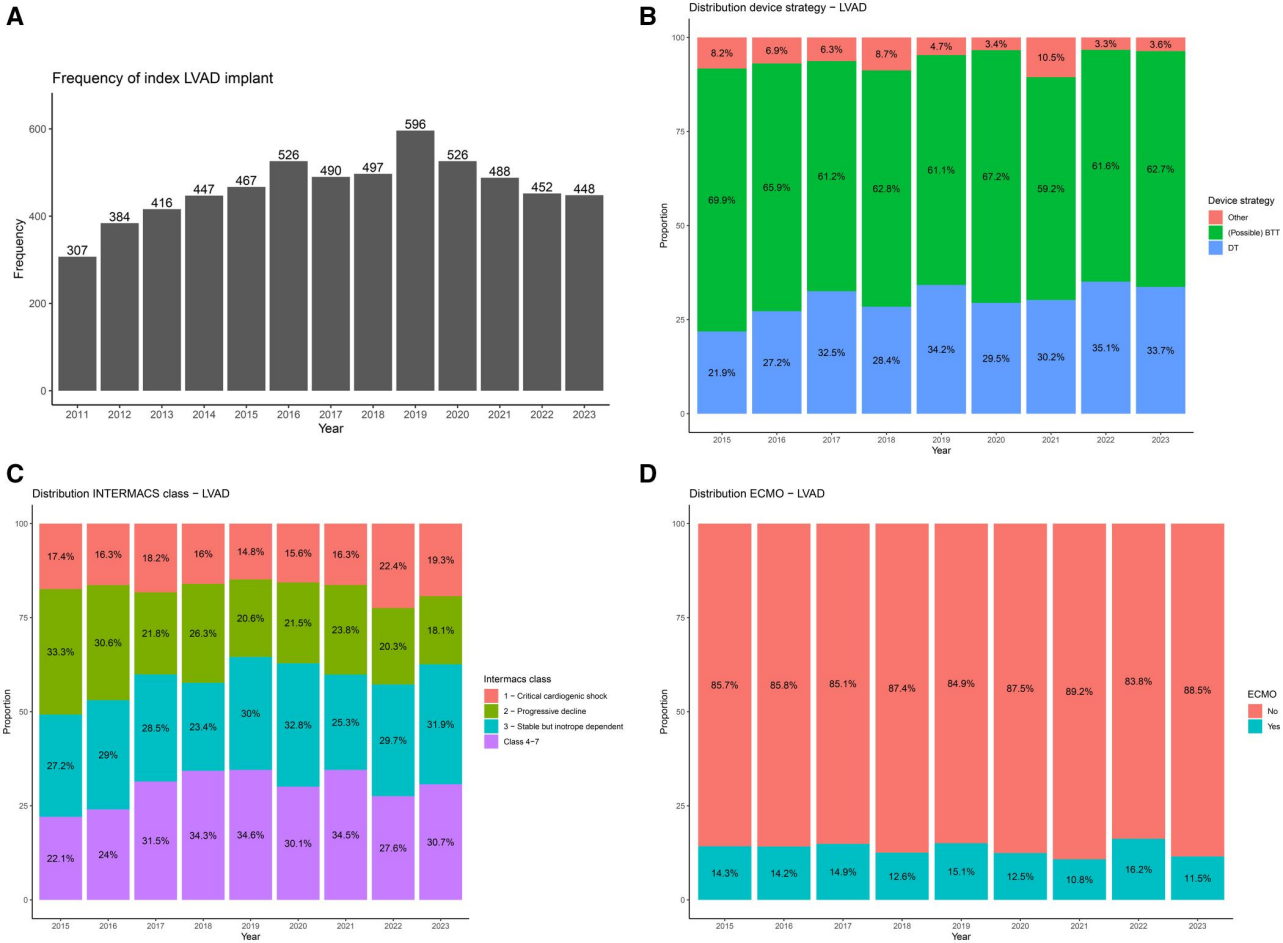
Distribution of device strategy (**A**), frequency of LVAD implantation class (**B**) device strategy, (**C**) INTERMACS class and (**D**) pre-LVAD ECMO BTT: bridge to transplant, DT: destination therapy; ECMO: extracorporeal membrane oxygenation.

Data of 8791 implants in 8151 patients who gave permission to the participating hospitals to share their data with EUROMACS were selected for this report. After applying exclusion criteria in total 6408 patients undergoing 6962 implants (457 underwent multiple implants), of which 6497 encompassed isolated LVAD (Left Ventricular Assist Device) implantations, were selected for analyses (Fig. 1). Date of surgery ranged from March 2006 to August 2024. The implantations of included patients were performed in 17 countries (median 105, IQR 49–477) coming from 32 centres (Fig. 2). Frequency of annual index implants is shown in Fig. 3A. In 2022, almost exclusively continuous fully magnetic flow devices were implanted (Supplementary Material, Fig. S1).

### Patient characteristics

Baseline variables of isolated LVAD (±temporary RVAD [Right Ventricular Assist Device]) procedures are presented in Table 1. Figure 3B–D presents relative change over time in device strategy (Fig. 3B), INTERMACS class (Fig. 3C) and pre-LVAD use of pre-LVAD extracorporeal membrane oxygenation (Fig. 3D).

**Table 1:** Baseline characteristics of isolated LVAD (±temporary RVAD) implants

Characteristics	
No. of procedures	6497
Patient demographics and comorbidities	
Age, median [IQR]	58.00 [49.00, 64.00]
Male, *n* (%)	5405 (83.2)
Non-ischaemic cardiomyopathy, *n* (%)	4161 (67.8)
INTERMACS profile, *n* (%)	
1: Critical cardiogenic shock	1051 (17.0)
2: Progressive decline	1660 (26.8)
3: Stable but inotrope dependent	1775 (28.7)
4–7: Resting symptoms—NYHA 3	1704 (27.5)
History neurological event	
Ischaemic CVA	426 (7.8)
ICB	40 (0.7)
TIA	177 (3.3)
Diabetes, *n* (%)	1667 (26.8)
Major MI	882 (15.8)
COPD, *n* (%)	623 (11.0)
Currently smoking, *n* (%)	714 (19.3)
Rhythm, *n* (%)	
Sinus	2745 (52.1)
Paced	1376 (26.1)
Atrial fibrillation	953 (18.1)
Atrial flutter	83 (1.6)
Other	111 (2.1)
Laboratory measurements, median [IQR]	
Haemoglobin	11.60 [9.83, 13.50]
BUN	44.55 [26.80, 72.78]
Bilirubin	1.20 [0.74, 2.02]
Creatinine	114.00 [88.00, 154.00]
Peri-implant characteristics	
Pre-LVAD ECLS, *n* (%)	767 (12.4)
Pre-LVAD IABP, *n* (%)	563 (10.0)
Temporary RVAD, *n* (%)	329 (5.1)
N IV inotropes, median [IQR]	1.00 [0.00, 2.00]
Device strategy, *n* (%)	
Possible bridge to transplant	2271 (35.3)
Bridge to transplant	1971 (30.6)
Destination therapy	1769 (27.5)
Rescue therapy	272 (4.2)
Bridge to recovery	138 (2.1)
Others	13 (0.2)
Hospital outcome	
Hospital deaths, *n* (%)	893 (14.6)
ICU/CCU stay (days), median [IQR]	10.00 [5.00, 24.00]

BUN: blood urea nitrogen; CCU: critical care unit; COPD: chronic obstructive pulmonary disease; CVA: cerebral vascular accident; ECLS: extra corporeal life support; IABP: intra-aortic balloon pump; ICB: intercranial bleeding; ICU: intensive care unit; TIA: transient ischaemic attack.

### Endpoints

In patients undergoing isolated LVAD implants, hospital mortality was 893 (14.6%). The crude probability of 30-day and 1-year mortality, major bleeding and ischaemic stroke in patients who received isolated LVAD implants is shown in Table 2.

**Table 2:** Crude 30-day and 1-year mortality, major bleeding and ischaemic stroke probabilities

	30 days			1 year		
	Probability (%)	Patients (*n*)	Centres (*n*)	Probability (%)	Patients (*n*)	Centres (*n*)
Mortality	9.6%	6172	32	27.1%	4456	20
Major bleeding	12.6%	5868	30	25.4%	4491	20
Ischaemic stroke	2.1%	5958	31	5.3%	4462	21

### Follow-up

**Figure 4:**
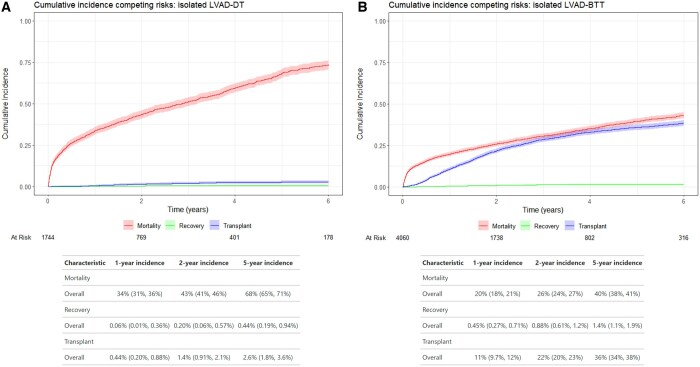
Cumulative incidence of mortality, recovery and transplant of DT (**A**) and BTT (**B**) patients. BTT: bridge to transplant; DT: destination therapy.

**Figure 5:**
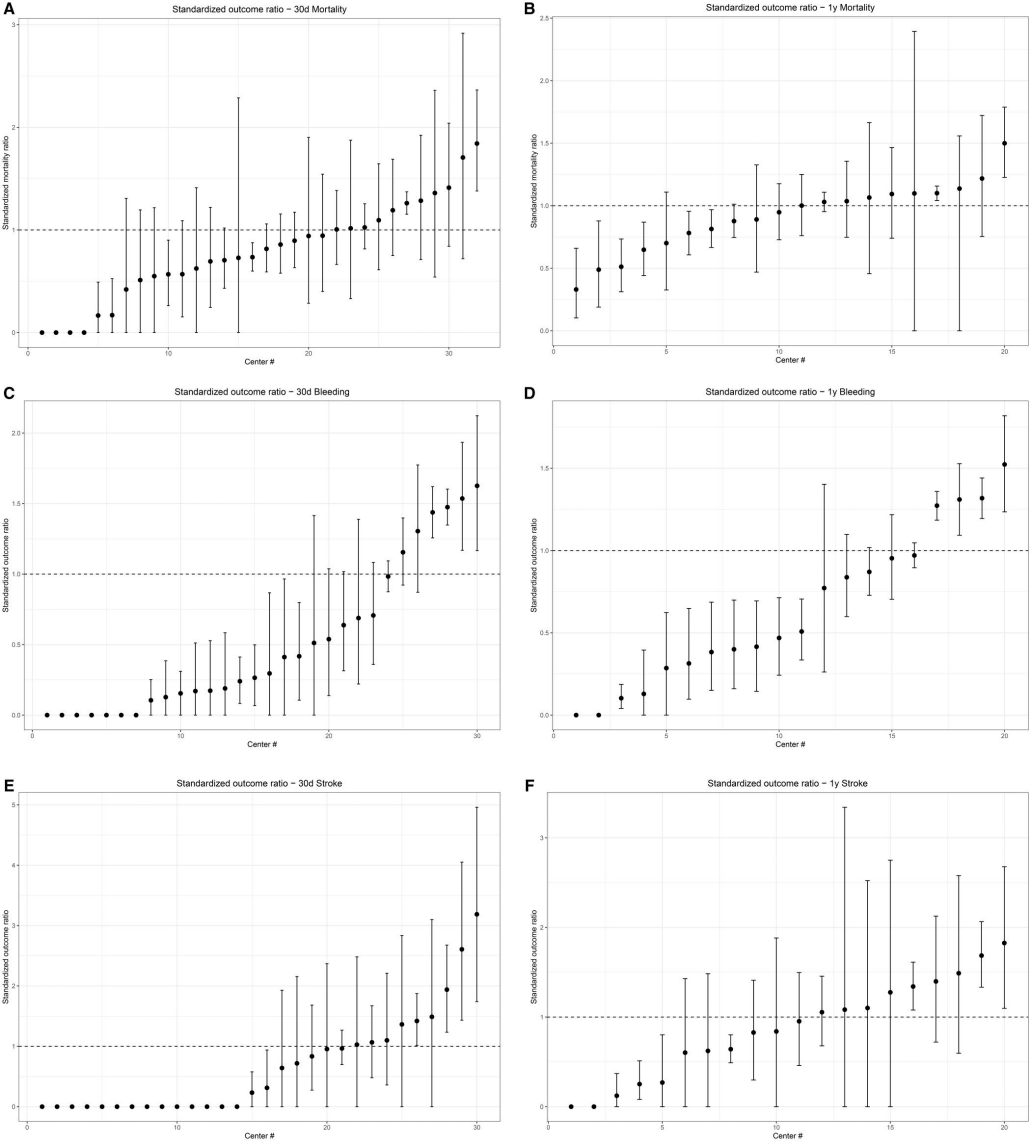
Standardized outcome ratio. (**A**) Standardized outcome ratio—30-day mortality. (**B**) Standardized outcome ratio—1-year mortality. (**C**) Standardized outcome ratio—30-day bleeding. (**D**) Standardized outcome ratio—1-year bleeding. (**E**) Standardized outcome ratio—30-day stroke. (**F**) Standardized outcome ratio—1-year stroke.

Median follow-up was 1.5 (IQR 0.4–3.4) years and follow-up completeness was 87% according to the Clark C completeness measure. Actual mortality for patients undergoing isolated LVAD implants with DT or bridge to transplant strategy is shown in Fig. 5A and B. In bridge to transplant patients, 2 years after implantation, a mortality of 26% (95% CI 24–27%) was observed, 22% (95% CI 20–23%) were transplanted and 0.9% (95% CI 0.6–1.2) had an explant due to recovery. In DT patients, 2-year mortality was 43% (95% CI 41–46%), 1.4% (95% CI 0.85–2.1%) were transplanted and 0.2% (0.1–0.6%) were explanted due to recovery (Fig. 4).

### Prediction of expected events

Coefficients of the penalized logistic regression models are shown in Supplementary Material, Table S4. The Area under the Curve of the 30-day and 1-year penalized logistic models for 30 day and 1-year event prediction for mortality, bleeding and ischaemic stroke is shown in Supplementary Material, Table S5. Most models had acceptable performance. Calibration plots are shown in Supplementary Material, Figs 3–8. The models were moderately to well calibrated, except for the model predicting 1-year ischaemic stroke, which underestimated stroke probability in higher-risk patients.

### Standardized outcome ratios

The standardized outcome ratios of 30-day and 1-year mortality, bleeding and ischaemic stroke are shown in Fig. 5. Figure 5 shows considerable variability among standardized outcome ratios among centres. In most centres the 95% confidence interval crosses the 1. Centres that that had a higher standardized outcome ratio for 30-day bleeding also have a higher standardized outcome ratio for 30-day ischaemic stroke (Spearman *r*: 0.42, *P* = 0.03). This was also true for 1-year ischaemic stroke and bleeding (Spearman *r*: 0.56, *P* = 0.008).

### Scientific output

Scientific output derived from the EUROMACS data increased with 10 new peer-reviewed publications published in 2022 and 5 publications in 2023 (Supplementary Material, Fig. S2).

## DISCUSSION

The 4th report from the EUROMACS registry demonstrates consistent annual registered implantations and scientific output utilizing the database. This report shows that major bleeding still poses a significant burden for patients. Standardized outcome ratios demonstrate considerable variability among centres.

INTERMACS reports a 27.9% actual mortality probability at 2 years for all DT patients on continuous flow devices implanted between 2015 and 2019 [8], whereas 2-year mortality for DT in EUROMACS was found to be 43%. Differences in long-term outcomes among registries worldwide (EUROMACS, INTERMACS) are multidimensional and contrast with comparable trial and trial-like registry outcomes (MOMENTUM versus ELEVATE), in which outcomes are comparable (74% survival MOMENTUM at 2 years versus 74.2% ELEVATE at 2 years) [1, 9]. One (of many) potential explanations might be the less strict patient selection in highly advanced and acute heart failure patients in Europe. Achievable outcomes in this patient group are generally less favourable, even after adjustment for observed patient differences. Since no other viable treatment option for these patients exists, a scientific and societal discussion is needed to further specify strategies in the future. Compared with other medical fields, this is a discussion that was also seen in oncology (with respect to treatment and success rates, and patient/physicians’ viewpoints on risk/benefits in different disease stages of various malignancies) [10, 11].

### Prediction of expected events

In this report, prediction models were developed to estimate the expected number of events. Most prediction models had acceptable performance. Yet, they do not reach the performance seen in risk scores in other cardiovascular disease domains [12, 13], underscoring the difficulty of event prediction in this group of patients. Of note, the aim of this report was not to develop parsimonious prediction models for outcome prediction.

### Standardized outcome ratios

Major bleeding and stroke after isolated LVAD implantation are still prevalent. Also, in the INTERMACS registry, 90-day major bleeding and stroke were reported to be 20% and 6%, respectively [14]. Centres that had higher standardized outcome ratio for 30-day and 1-year major bleeding also had higher standardized outcome ratios for 30 day and 1-year ischaemic stroke. The interplay between bleeding and thromboembolic stroke has been described previously, with an increased risk of thromboembolic events after a bleeding event [15, 16]. Major infection is most likely also an important mediator, as EUROMACS data showed that a major infection increases the hazard of both bleeding events and strokes [17]. Other mechanisms may include difficulty stabilizing INR (International Normalized Ratio) during follow-up and/or the use of pro-coagulant treatments and blood cell transfusions in case of bleeding following thromboembolic events. In this study, we did not conduct volume–outcome analyses, as a comprehensive analysis using EUROMACS data was recently published [18].

### Future perspectives of quality improvement in mechanical circulatory support

As recommended by the 2023 International Society of Heart Lung Transplant guidelines for patients with MCS, each centre implanting durable MCS devices should have a dedicated quality assurance and improvement program (level of evidence: C) [14]. The QUIP Committee of EACTS endorses this recommendation. The EUROMACS registry may serve as a European benchmark for these efforts. To continue being a relevant benchmark, the EUROMACS registry outcome definitions will be updated according to the latest INTERMACS 2020 definitions [19]. The ability to track quality of life using Patient Reported Outcome Measures (PROMs) is already feasible through the longitudinal utilization of EQ-5D [20]. In addition, capturing of additional recommended PROMs within the EUROMACS registry is supported on request.

### Limitations

Contrary to registries in other parts of the world, participation in EUROMACS is not mandatory. Therefore, surveillance and improvement of data quality are ongoing efforts. Like other multicentre international registries, EUROMACS is faced with missing data, incomplete follow-up and potential selection bias. We only included centres with 90% naïve completeness in the standardized outcome ratios. Patients who did not have 1-year follow-up were excluded from these analyses; hence the standardized outcomes ratios do not reflect the most recent patient sample. Nevertheless, calculating expected events by prediction models is still a superior approach than using the overall probability as expected outcome probability, as prediction models do consider differences in patient characteristics. Additionally, in case follow-up, completeness is associated with worse centre performance, this may bias results of the overall estimates. This registry is limited to patients who received durable MCS, and no inference can be made on risk avoidance and acceptance.

## CONCLUSIONS

Most included centres perform as expected given the demographics and comorbidities of patients. A positive correlation was found between standardized bleeding and ischaemic stroke ratios, reflecting the need for continuously monitoring of adverse events by quality improvement programmes.

## Supplementary Material

ezaf016_Supplementary_Data

## Data Availability

Data are available on request to the Scientific Committee of EUROMACS.

## ABBREVIATIONS

DT: Destination therapy
EACTS: European Association for Cardio-Thoracic Surgery
EUROMACS: European Registry for Patients with Mechanical Circulatory Support
INTERMACS: Interagency Registry for Mechanically Assisted Circulatory Support
MCS: Mechanical circulatory support
PROM: Patient Reported Outcome Measure
QUIP: Quality Improvement Program
