# Supplementary material for: The fourth report of the European Registry for Patients with Mechanical Circulatory Support (EUROMACS) of the European Association for Cardiothoracic Surgery: focus on standardized outcome ratios
Source: Eur J Cardiothorac Surg. 2025 Jan 28;67(2):ezaf016. doi: 10.1093/ejcts/ezaf016 (PMC11879288; doi:10.1093/ejcts/ezaf016)

Contents

[Supplementary text 1 2](#_Toc184918342)

[Supplementary Table 1 3](#_Toc184918343)

[Supplementary Table 2 4](#_Toc184918344)

[Supplementary Table 3: 6](#_Toc184918345)

[Supplementary Table 4 8](#_Toc184918346)

[Supplementary Table 5: 11](#_Toc184918347)

[Supplementary Figure 1 12](#_Toc184918348)

[Supplementary Figure 2 13](#_Toc184918349)

[Supplementary Figure 3 14](#_Toc184918350)

[Supplementary Figure 4 15](#_Toc184918351)

[Supplementary Figure 5 16](#_Toc184918352)

[Supplementary Figure 6 17](#_Toc184918353)

[Supplementary Figure 7 18](#_Toc184918354)

[Supplementary Figure 8 19](#_Toc184918355)

Supplementary text 1: Detailed analyses

*Multiple Imputation*

Baseline variables <40% missing variables were imputed using multiple imputation with chained equations. Percentage of missing values and if they are utilized in the multiple imputation is shown in Supplementary Table 2. Correlation between variables was investigated using Spearman’s R. In case 2 variables were highly correlated; only the variable with least missing values was used in the imputation models. Five imputed datasets were generated using 15 iterations each. Convergence was investigated using convergence plots and imputed values were investigated using density plots. All analyses were done on the separated datasets and pooled according to Rubin’s rules. The statistical package “mice” in R was used for the imputations.

*Logistic Prediction models*

In total 60 variables were used in the penalized logistic models (Supplementary Table 1). Area under curve confidence interval was derived based on bootstrapping using 1000 resamples. The calibration plots in Supplementary Figure 3-8 are based on the first imputed dataset. The package “glmnet” and “pROC” in R were used to derive the prediction models and Area under the curve.

Supplementary Table 1: Follow-up completeness of excluded centers

| **Center** | **Follow-up completeness (Clark C)** |
| --- | --- |
| 1 | 32.29 % |
| 2 | 1.53 % |
| 3 | 27.27 % |
| 4 | 0.03 % |
| 5 | 56.85 % |
| 6 | 0.47 % |
| 7 | 59.88 % |
| 8 | 57.79 % |
| 9 | 0.03 % |
| 10 | 15.02 % |
| 11 | 17.59 % |
| 13 | 20.29 % |
| 14 | 44.07 % |
| 15 | 25.56 % |
| 16 | 8.19 % |
| 17 | 10.46 % |

Supplementary Table 2: Variables used in the penalized logistic regression

| **Predictor** | **Format** |
| --- | --- |
| Age | Continuous |
| Body surface index | Continuous |
| Blood urea | Continuous |
| Total bilirubin | Continuous |
| Creatinine | Continuous |
| CRP | Continuous |
| Sodium | Continuous |
| Potassium | Continuous |
| ALAT | Continuous |
| LDH | Continuous |
| White blood cel count | Continuous |
| Hemoglobin | Continuous |
| Platelets | Continuous |
| INR | Continuous |
| PTT | Continuous |
| Number of inotropes | Continuous |
| Sex | Categorical |
| Ethnic origin | Categorical |
| Primary diagnosis | Categorical |
| Intermacs profile | Categorical |
| Current device strategy | Categorical |
| Time since first diagnosis | Categorical |
| ICD | Categorical |
| Ascitis | Categorical |
| Major MI | Categorical |
| Cardiac surgery | Categorical |
| Positive blood cultures | Categorical |
| Other concomitant surgical procedures | Categorical |
| Major infections | Categorical |
| Peripheral oedema | Categorical |
| Symptomatic Peripheral Vascular Disease | Categorical |
| History Neurological Event" | Categorical |
| Carotid Artery Disease | Categorical |
| Diabetes | Categorical |
| Bloodtype | Categorical |
| Rhesusfactor | Categorical |
| DeviceType | Categorical |
| Device Brand LVAD | Categorical |
| Rhythm | Categorical |
| Mitralregurgitation | Categorical |
| Tricuspid regurgitation | Categorical |
| Aortic regurgitation | Categorical |
| EF grade | Categorical |
| Dialysis | Categorical |
| Intubation | Categorical |
| Previous VAD | Categorical |
| Ultrafiltration | Categorical |
| Ventilator | Categorical |
| Feeding Tube | Categorical |
| ECMO | Categorical |
| IABP | Categorical |
| ARBs | Categorical |
| Amiodarone | Categorical |
| ACE inhibitors | Categorical |
| Betablockers | Categorical |
| Aldosterone antagonist | Categorical |
| Loopdiuretics | Categorical |
| Anticoagulant therapy | Categorical |
| Nitric Oxide | Categorical |
| Year | Continuous |

Supplementary Table 3:

| **Variable** | **Missing (%)** | **Included in multiple imputation** |
| --- | --- | --- |
| Age | 0,0 | Yes |
| Gender | 0,0 | Yes |
| Blood type | 0,1 | Yes |
| Rhesus factor | 0,8 | Yes |
| Device Type | 1,1 | Yes |
| Current Device Strategy | 2,9 | Yes |
| Device Brand LVAD | 6,2 | Yes |
| Pulsatile Device | 6,2 | No |
| Diabetes | 6,5 | Yes |
| INTERMACS Patient Profile | 6,8 | Yes |
| Dialysis | 7,3 | Yes |
| LVAD Type Of Driving | 7,3 | No |
| ECMO | 7,3 | Yes |
| Primary Diagnosis | 7,8 | Yes |
| Patient Discharged To | 8,0 | No |
| Other VAD | 8,4 | Yes |
| Symptomatic Peripheral Vascular Disease | 11,2 | Yes |
| ICU-CCU Stay | 11,8 | No |
| BSA | 12,0 | Yes |
| Intubation | 14,7 | Yes |
| COPD | 14,8 | Yes |
| IABP | 15,2 | Yes |
| Major Infections | 15,4 | Yes |
| Cardiac Surgery | 15,5 | Yes |
| Major MI | 15,8 | Yes |
| Time since first cardiac diagnosis | 16,2 | Yes |
| White blood cel count | 16,9 | Yes |
| Other Surgical Procedures | 17,1 | Yes |
| Ultrafiltration | 17,4 | Yes |
| Ventilator | 17,6 | Yes (not as predictor) |
| History Of Neurological Event | 18,1 | Yes |
| Feeding Tube | 18,6 | Yes |
| INR | 18,8 | Yes |
| Hemoglobin | 19,1 | Yes |
| Nitric Oxide On Admission | 19,7 | Yes |
| Platelet | 20,3 | Yes |
| ECG rhythm | 20,8 | Yes |
| ICD device | 21,5 | Yes |
| Amiodarone | 21,8 | Yes |
| Loop diuretics | 22,1 | Yes |
| Number intropes | 22,1 | Yes |
| Sodium | 22,9 | Yes |
| Tricuspid regurgitation | 23,2 | Yes |
| Aldosterone antagonist | 23,8 | Yes |
| Betablockers | 23,9 | Yes |
| Potassium | 23,9 | Yes |
| ACE inhibitors | 24,1 | Yes |
| CRPC reactive protein | 24,2 | Yes |
| Total bilirubin | 24,8 | Yes |
| ARBO | 25,1 | Yes |
| Anticoagulant therapy | 26,2 | Yes |
| Aortic regurgitation | 27,6 | Yes |
| Ethnic origin | 27,7 | Yes |
| Volume Status peripheral edema | 28,6 | Yes |
| Positive Blood Cultures | 28,9 | Yes |
| PTT | 29,5 | Yes |
| Mitral regurgitation | 30,5 | Yes |
| Creatinine | 30,9 | Yes |
| Carotid Artery Disease | 33,7 | Yes |
| EF Grade | 33,9 | Yes |
| LDH | 34,7 | Yes |
| Blood Urea Nitrogen | 34,8 | Yes |
|  |  |  |

Supplementary Table 4: Coefficient and hyperparameter of Lasso Logistic regression models

| Predictor | Mortality (30d) | Mortality (1y) | Bleeding (30d) | Bleeding (1y) | Stroke (30d) | Stroke (1y) |
| --- | --- | --- | --- | --- | --- | --- |
| (Intercept) | 65.480 | -2.013 | -2.324 | -54.629 | -3.666 | -3.636 |
| Age | 0.022 | 0.022 | 0.006 | 0.009 | 0.000 | 0.000 |
| BSA | 0.062 | 0.196 | -0.019 | -0.062 | -0.300 | 0.000 |
| Blood Urea Nitrogen | 0.002 | 0.003 | 0.000 | 0.000 | 0.000 | 0.000 |
| Total bilirubin | 0.012 | 0.014 | 0.024 | 0.020 | 0.000 | 0.000 |
| Creatinine | 0.001 | 0.000 | 0.002 | 0.001 | 0.000 | 0.000 |
| CRPC reactive protein | 0.001 | 0.000 | -0.002 | -0.001 | 0.000 | -0.001 |
| Sodium | -0.002 | -0.008 | 0.000 | -0.002 | 0.000 | 0.006 |
| Potassium | -0.006 | -0.018 | 0.033 | 0.001 | 0.004 | 0.000 |
| LDH | 0.0002 | 0.000 | 0.000 | 0.000 | 0.000 | 0.000 |
| WBC | 0.023 | 0.010 | 0.000 | -0.001 | 0.007 | 0.000 |
| Hemoglobin | 0.000 | 0.000 | -0.003 | -0.006 | 0.000 | 0.000 |
| Platelet | -0.001 | -0.001 | 0.000 | 0.000 | 0.000 | 0.000 |
| INR | 0.007 | 0.013 | -0.007 | 0.000 | -0.064 | -0.075 |
| PTT | 0.005 | 0.007 | 0.006 | 0.004 | 0.000 | 0.000 |
| Number intropes | 0.004 | 0.064 | 0.089 | 0.077 | 0.000 | -0.018 |
| Gender - male | -0.139 | -0.133 | -0.049 | 0.000 | 0.000 | 0.000 |
| Ethnic origin - Asian | 0.000 | -0.028 | -0.350 | 0.107 | -0.059 | 0.037 |
| Ethnic origin - Caucasian | 0.000 | -0.035 | 0.000 | 0.000 | 0.198 | 0.017 |
| Ethnic origin - Hawaiian or other pacific islander | 0.000 | -0.121 | 0.000 | 0.176 | 0.000 | 0.000 |
| Ethnic origin - Other | 0.071 | 0.000 | 0.053 | 0.000 | 0.000 | -0.020 |
| Primary Diagnosis - Non ischemic | 0.000 | -0.064 | -0.010 | 0.000 | 0.000 | -0.104 |
| INTERMACSPatientProfile - 2 - Progressive decline (vs 1) | -0.128 | 0.000 | 0.000 | 0.000 | 0.000 | -0.082 |
| INTERMACSPatientProfile - 3 - Stable but inotrope dependent (vs 1) | -0.433 | -0.339 | -0.211 | -0.027 | -0.009 | 0.000 |
| INTERMACSPatientProfile 4 to 6 - Resting symptoms (vs 1) | -0.419 | -0.226 | -0.120 | 0.000 | 0.007 | 0.214 |
| Current Device Strategy - Bridge to transplant (patient currently listed for transplant) | 0.000 | -0.073 | 0.012 | 0.000 | 0.000 | 0.000 |
| Current Device Strategy - Destination therapy | 0.281 | 0.474 | 0.066 | 0.038 | 0.000 | -0.015 |
| Current Device Strategy - Other | 0.158 | -0.272 | 0.000 | 0.000 | 0.000 | 0.000 |
| Current Device Strategy - Possible bridge to transplant | 0.000 | 0.000 | -0.105 | -0.173 | 0.000 | 0.000 |
| Current Device Strategy - Rescue therapy | 0.802 | 0.670 | -0.020 | -0.201 | 0.000 | 0.000 |
| Time since first cardiac diagnosis - One month to a year | -0.054 | -0.102 | 0.000 | 0.001 | 0.000 | 0.000 |
| Time since first cardiac diagnosis - One to two years | 0.000 | 0.000 | 0.000 | -0.009 | 0.000 | 0.000 |
| Time since first cardiac diagnosis - Over two years | 0.000 | 0.085 | 0.000 | 0.000 | 0.000 | 0.000 |
| ICD | 0.000 | 0.000 | 0.000 | 0.000 | -0.004 | 0.000 |
| COPD | -0.049 | 0.000 | 0.000 | 0.089 | 0.000 | 0.000 |
| Major MI | 0.012 | 0.000 | 0.000 | -0.010 | 0.000 | 0.000 |
| Cardiac Surgery | 0.022 | 0.286 | 0.000 | 0.004 | 0.000 | -0.008 |
| Positive Blood Cultures | 0.013 | 0.025 | 0.000 | -0.076 | 0.022 | 0.000 |
| Other Surgical Procedures | 0.000 | 0.000 | -0.005 | 0.000 | 0.000 | 0.000 |
| Major Infections | 0.000 | 0.008 | 0.282 | 0.219 | 0.357 | 0.000 |
| Volume Status peripheral edema - Mild | -0.004 | 0.000 | 0.043 | 0.052 | -0.061 | -0.034 |
| VolumeStatusperipheraledema - Moderate | 0.175 | 0.157 | 0.011 | 0.003 | 0.000 | 0.000 |
| Volume Status peripheral edema - Severe | 0.000 | 0.134 | 0.580 | 0.493 | 0.000 | 0.034 |
| Symptomatic Peripheral Vascular Disease | 0.132 | 0.239 | 0.008 | 0.057 | 0.005 | 0.000 |
| Neurological Event - ICB | -0.144 | 0.000 | -0.192 | 0.000 | 0.000 | 0.000 |
| Neurological Event - None | -0.038 | -0.040 | -0.169 | -0.023 | 0.116 | 0.000 |
| Neurological Event - TIA | -0.013 | 0.000 | -0.091 | 0.000 | 0.000 | 0.000 |
| Carotid Artery Disease | -0.003 | 0.002 | 0.017 | 0.120 | -0.083 | 0.105 |
| Diabetes | -0.015 | 0.003 | 0.000 | 0.005 | 0.132 | 0.003 |
| Bloodtype - AB | 0.000 | 0.055 | 0.000 | -0.002 | 0.000 | 0.000 |
| Bloodtype - B | -0.028 | -0.027 | -0.001 | 0.000 | 0.000 | 0.000 |
| Bloodtype - O | 0.000 | 0.000 | 0.000 | 0.000 | 0.052 | 0.159 |
| Rhesus factor - Positive | 0.000 | 0.000 | 0.000 | 0.000 | 0.000 | 0.000 |
| Device Type - LVAD | -1.492 | 0.000 | -0.735 | 0.000 | 0.000 | 0.000 |
| Device Type - LVAD.BiVAD | 0.000 | 0.000 | 0.000 | 0.000 | 0.000 | 0.000 |
| Device Type - LVAD.RVAD | 0.050 | 1.174 | 0.019 | 0.368 | 0.000 | 0.000 |
| Device Type - RVAD | 1.622 | 0.000 | 2.079 | 0.000 | 0.000 | 0.000 |
| Device Type - SVAD | 0.000 | 0.000 | 0.000 | 0.000 | 0.000 | 0.000 |
| DeviceType - Total artificial heart | 0.000 | 0.000 | 0.000 | 0.000 | 0.000 | 0.000 |
| Device Brand LVAD - HeartMate II LVAS | 0.000 | -0.067 | 0.000 | -0.004 | 0.000 | 0.000 |
| Device Brand LVAD HeartWare HVAD | 0.001 | 0.000 | 0.136 | 0.197 | 0.000 | 0.115 |
| Device Brand LVAD - Thoratec - HeartMate III | -0.061 | -0.320 | -0.013 | 0.000 | 0.000 | -0.347 |
| Atrial flutter | 0.227 | 0.000 | 0.000 | -0.040 | 0.115 | 0.144 |
| Other rhythm | 0.034 | -0.032 | -0.028 | 0.078 | 0.000 | 0.000 |
| Paced | 0.000 | 0.003 | 0.078 | 0.008 | 0.000 | 0.000 |
| Sinus rhythm | -0.034 | -0.184 | -0.053 | -0.016 | 0.000 | 0.019 |
| Mitral regurgitation - Trivial | 0.120 | 0.170 | 0.000 | -0.015 | 0.009 | 0.079 |
| Mitral regurgitation - Mild | 0.067 | 0.000 | 0.000 | 0.000 | 0.000 | 0.000 |
| Mitral regurgitation - Moderate | 0.000 | -0.102 | 0.000 | -0.018 | 0.079 | 0.004 |
| Mitral regurgitation - Severe | -0.080 | -0.085 | 0.035 | -0.001 | 0.000 | -0.015 |
| Tricuspid regurgitation - Trivial | 0.006 | -0.073 | 0.000 | 0.000 | 0.019 | 0.028 |
| Tricuspid regurgitation - Mild | -0.074 | -0.017 | -0.060 | 0.009 | 0.000 | 0.000 |
| Tricuspid regurgitation – Moderate | 0.000 | 0.003 | 0.000 | 0.006 | 0.000 | -0.024 |
| Tricuspid regurgitation - Severe | 0.216 | 0.112 | 0.151 | 0.146 | -0.344 | -0.099 |
| Aortic regurgitation - Trivial | 0.000 | -0.101 | -0.014 | 0.000 | -0.071 | -0.018 |
| Aortic regurgitation - Mild | 0.030 | 0.021 | 0.021 | 0.040 | 0.000 | 0.000 |
| Aortic regurgitation - Moderate | 0.007 | 0.224 | 0.000 | 0.000 | -0.125 | -0.092 |
| Aortic regurgitation - Severe | -0.020 | -0.228 | 0.037 | 0.026 | 0.000 | 0.000 |
| EF Grade 20-29% | 0.000 | 0.013 | 0.018 | 0.013 | 0.018 | 0.000 |
| EF Grade 30-39% | -0.005 | 0.021 | 0.177 | 0.218 | 0.000 | 0.007 |
| EF Grade 40-50% | 0.254 | 0.498 | 0.100 | 0.056 | 0.000 | -0.058 |
| EF Grade Over 50% | 0.449 | 0.492 | 0.015 | 0.000 | 0.000 | -0.074 |
| Dialysis | 0.039 | 0.275 | 0.042 | 0.087 | 0.000 | 0.000 |
| Intubation | 0.234 | 0.185 | -0.045 | -0.059 | 0.063 | 0.000 |
| Other VAD | 0.000 | -0.032 | -0.004 | 0.000 | 0.000 | 0.000 |
| Ultrafiltration | 0.000 | 0.026 | 0.230 | 0.255 | 0.005 | 0.000 |
| Ventilator | 0.000 | 0.000 | 0.000 | 0.000 | 0.060 | 0.020 |
| Feeding Tube | 0.000 | -0.007 | -0.245 | -0.053 | -0.205 | -0.085 |
| ECMO | 0.143 | 0.184 | 0.004 | 0.000 | 0.000 | 0.000 |
| IABP | 0.038 | -0.025 | -0.076 | -0.047 | 0.011 | 0.000 |
| ARB | 0.000 | 0.016 | 0.000 | -0.008 | 0.000 | 0.034 |
| Amiodarone – No | 0.000 | 0.010 | 0.000 | 0.016 | 0.000 | 0.050 |
| ACE inhibitors - No | 0.081 | 0.009 | 0.137 | 0.037 | 0.024 | 0.000 |
| Betablockers - No | 0.033 | 0.013 | 0.008 | 0.000 | 0.000 | 0.000 |
| Aldosterone antagonist - No | 0.000 | 0.139 | 0.154 | 0.015 | 0.000 | 0.036 |
| Loop diuretics - No | 0.173 | 0.334 | 0.038 | 0.145 | 0.000 | 0.000 |
| Anticoagulant therapy - No | -0.189 | -0.039 | -0.059 | 0.000 | 0.000 | 0.000 |
| Nitric Oxide - Yes | -0.082 | -0.077 | 0.808 | 0.519 | 0.015 | 0.411 |
| Year of intervention | -0.034 | 0.000 | 0.000 | 0.026 | 0.000 | 0.000 |
| *Hyperparameters* | | | | | | |
| Lambda | 0.003264 | 0.004201 | 0.1255965 | 0.2549544 | 0.00374 | 0.05289 |

Supplementary Table 5: Area under the curve of penalized logistic regression models**.**

|  | **30-days (AUC [95%CI])** | **1-year (AUC [95%CI])** |
| --- | --- | --- |
| **Mortality** | 76.8% (74.8% - 78.9%) | 74.7% (73.0% - 76.3%) |
| **Bleeding** | 73.4% (71.5% - 75.3%) | 68.2% (66.4% - 70.0%) |
| **Ischemic stroke** | 70.2% (65.6% - 74.4%) | 67.7% (64.1% - 71.4%) |

AUC: Area under the curve, CI: Confidence interval

Supplementary Figure 1: Stacked barplot of device type

 
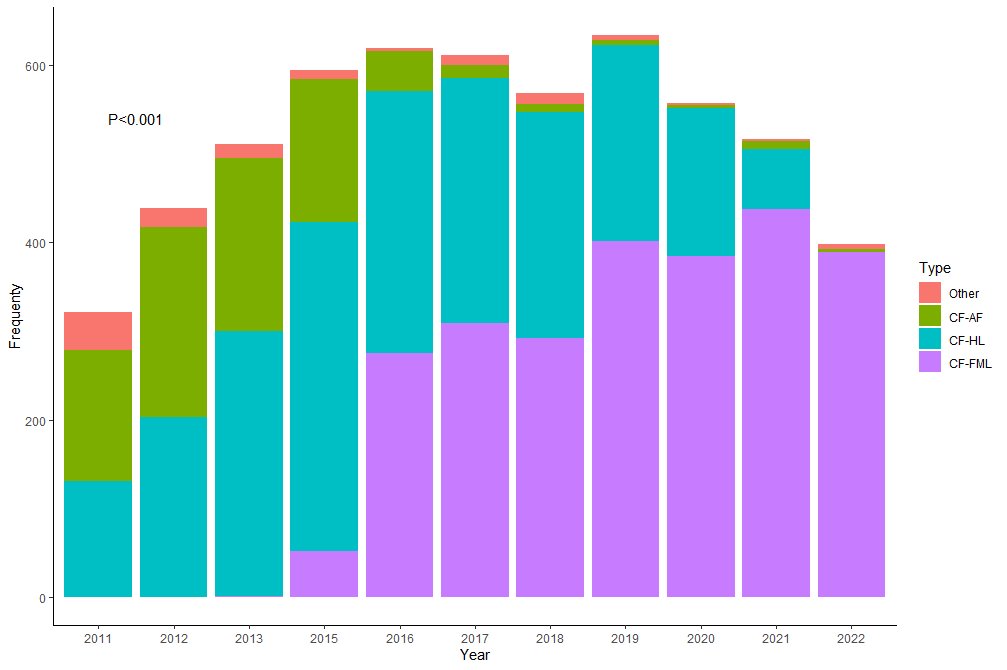


Supplementary Figure 2: Publications and yearly cumulative impact factor

 
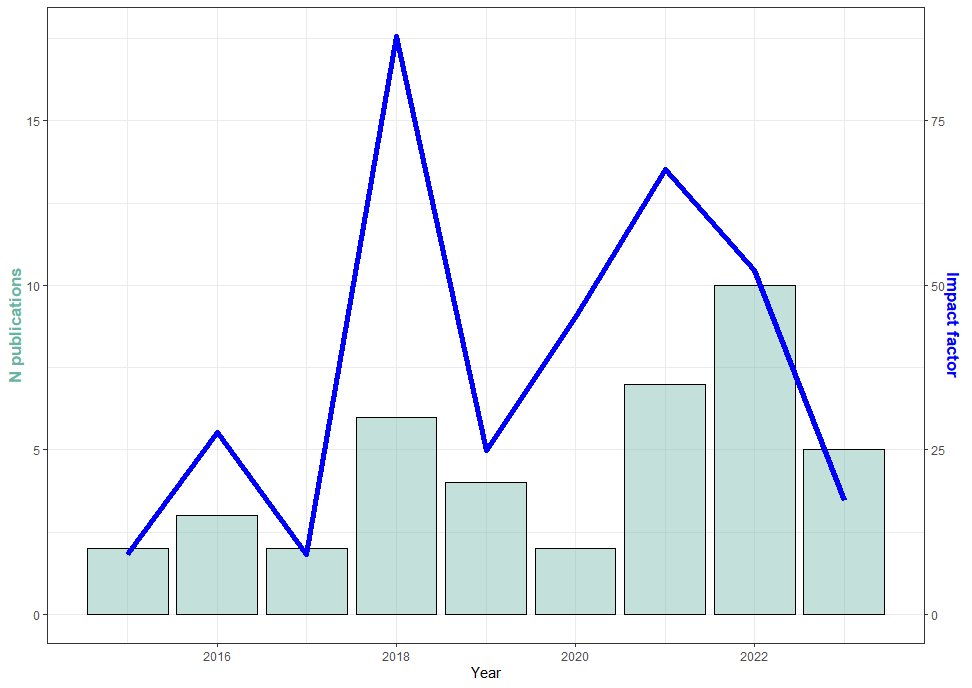


Supplementary Figure 3: Calibration plot 30d mortality


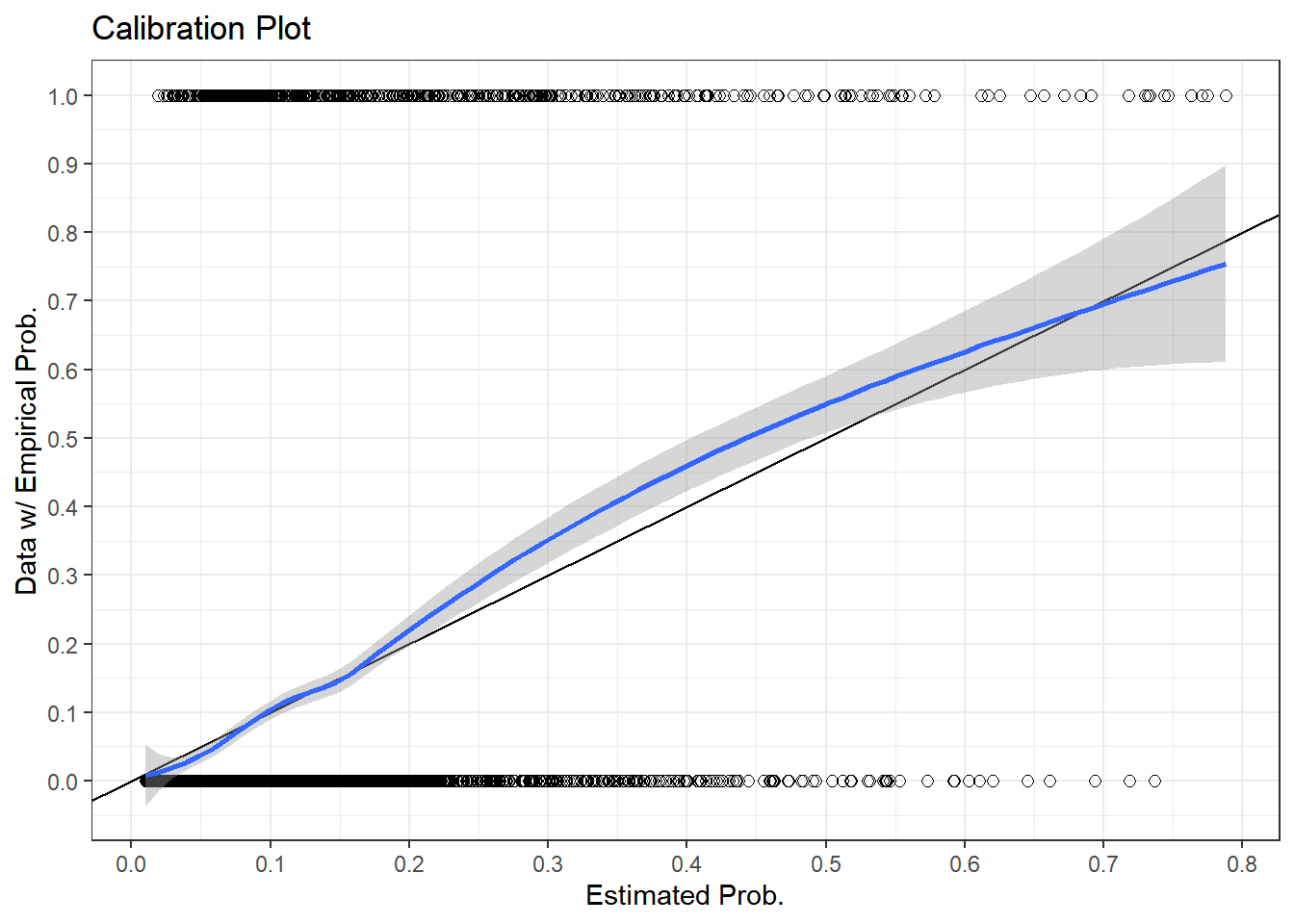


Supplementary Figure 4: Calibration plot 1y mortality


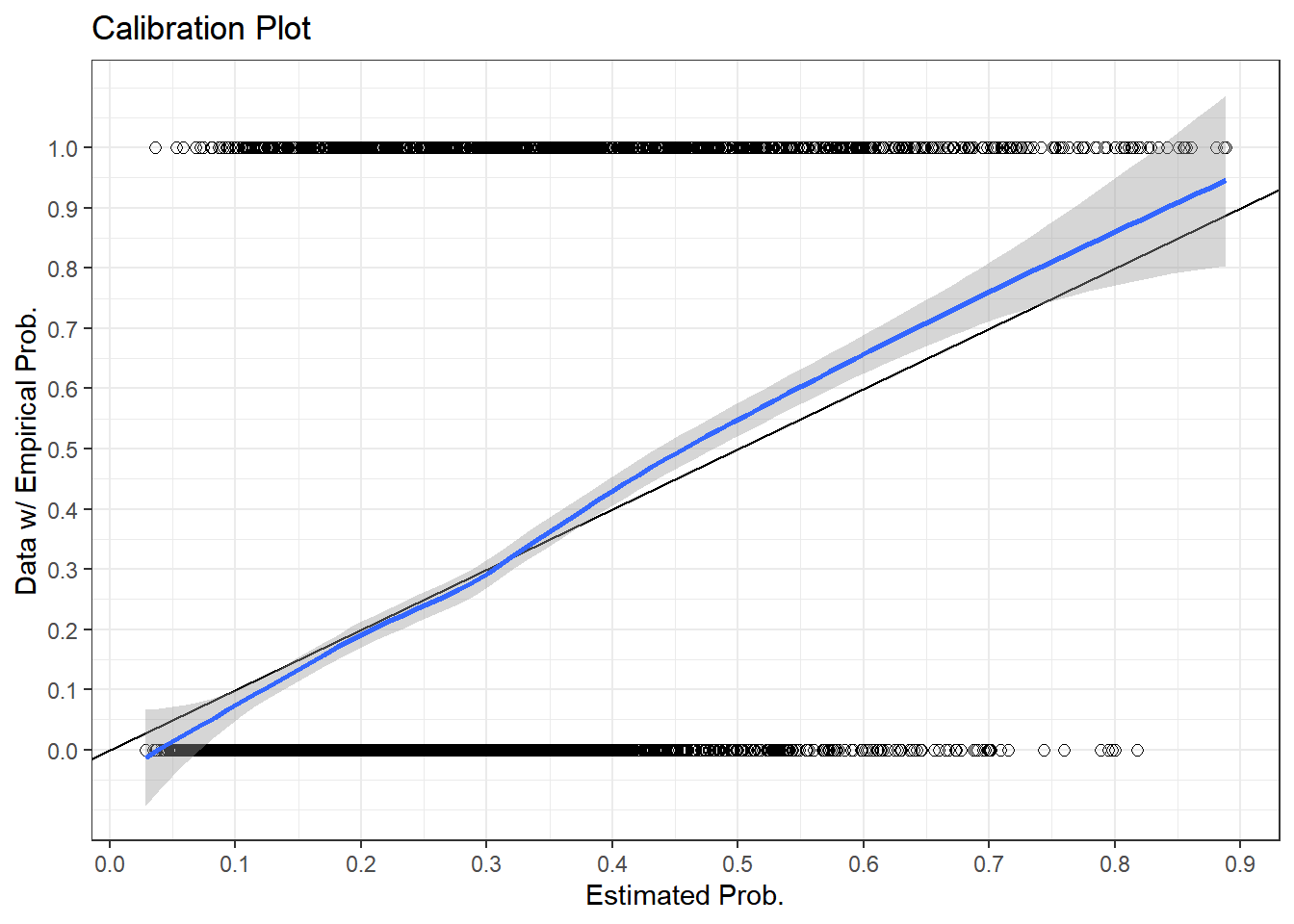


Supplementary Figure 5: Calibration plot 30d Bleeding


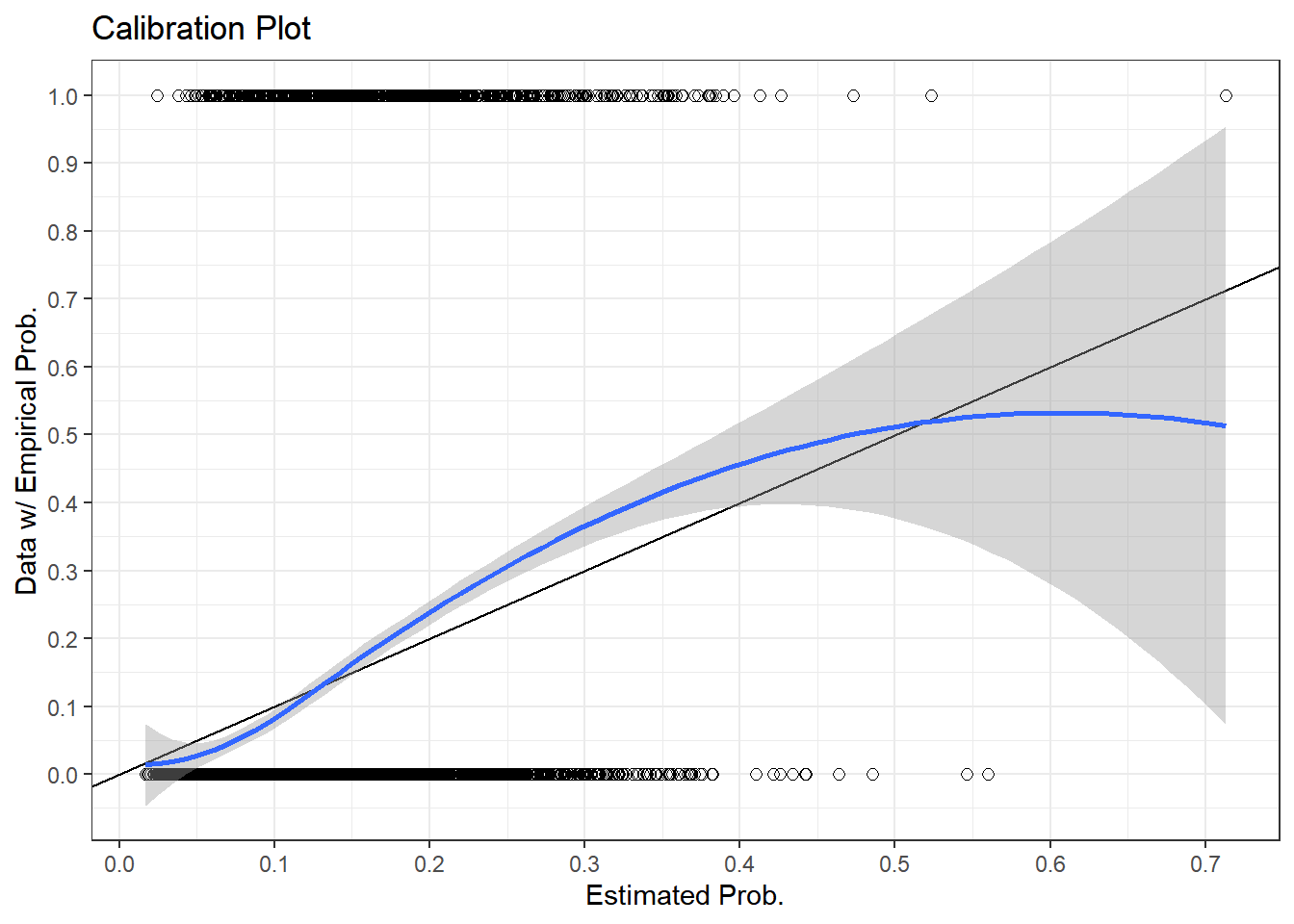


Supplementary Figure 6: Calibration plot 1y Bleeding


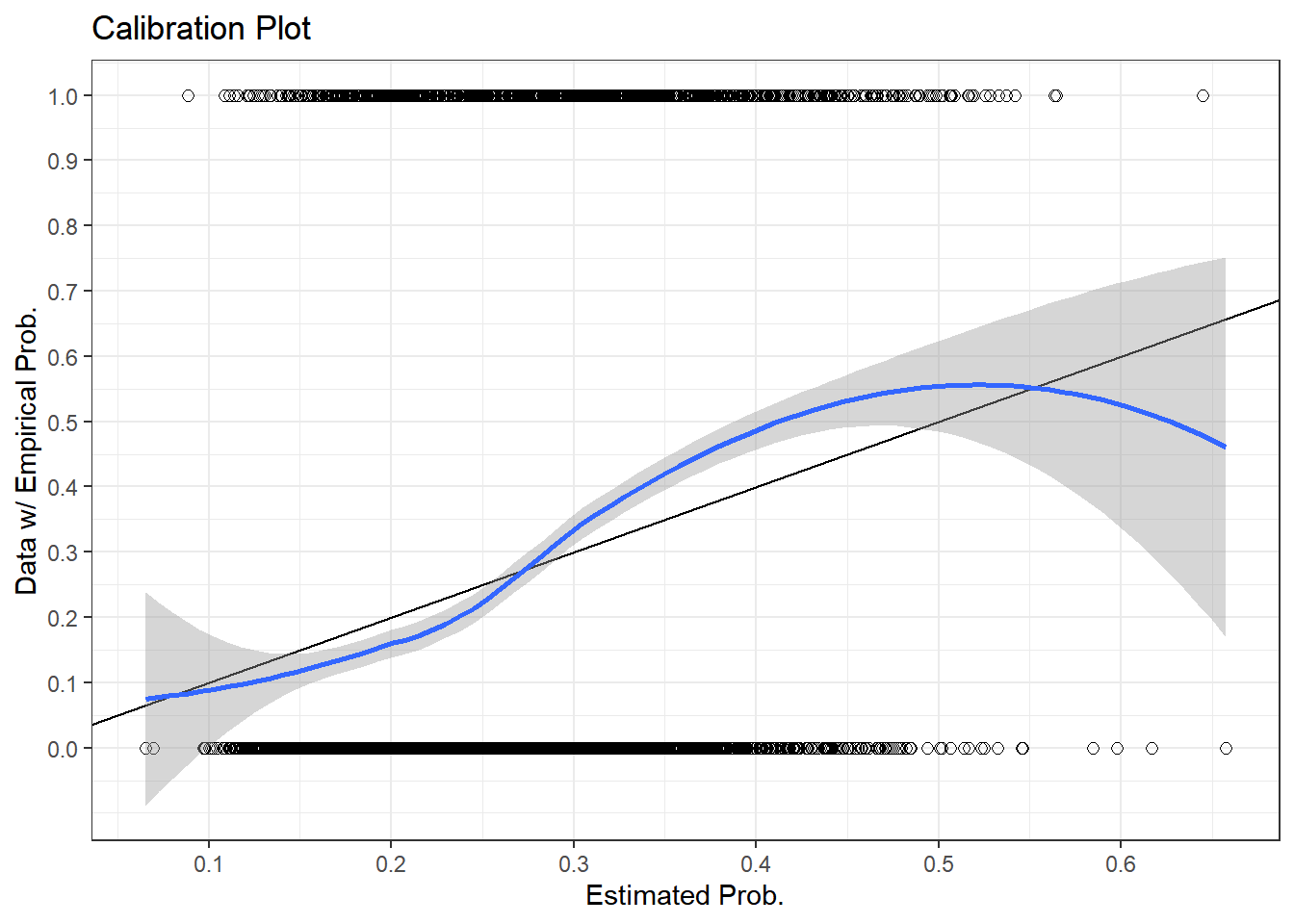


Supplementary Figure 7: Calibration plot 30d stroke


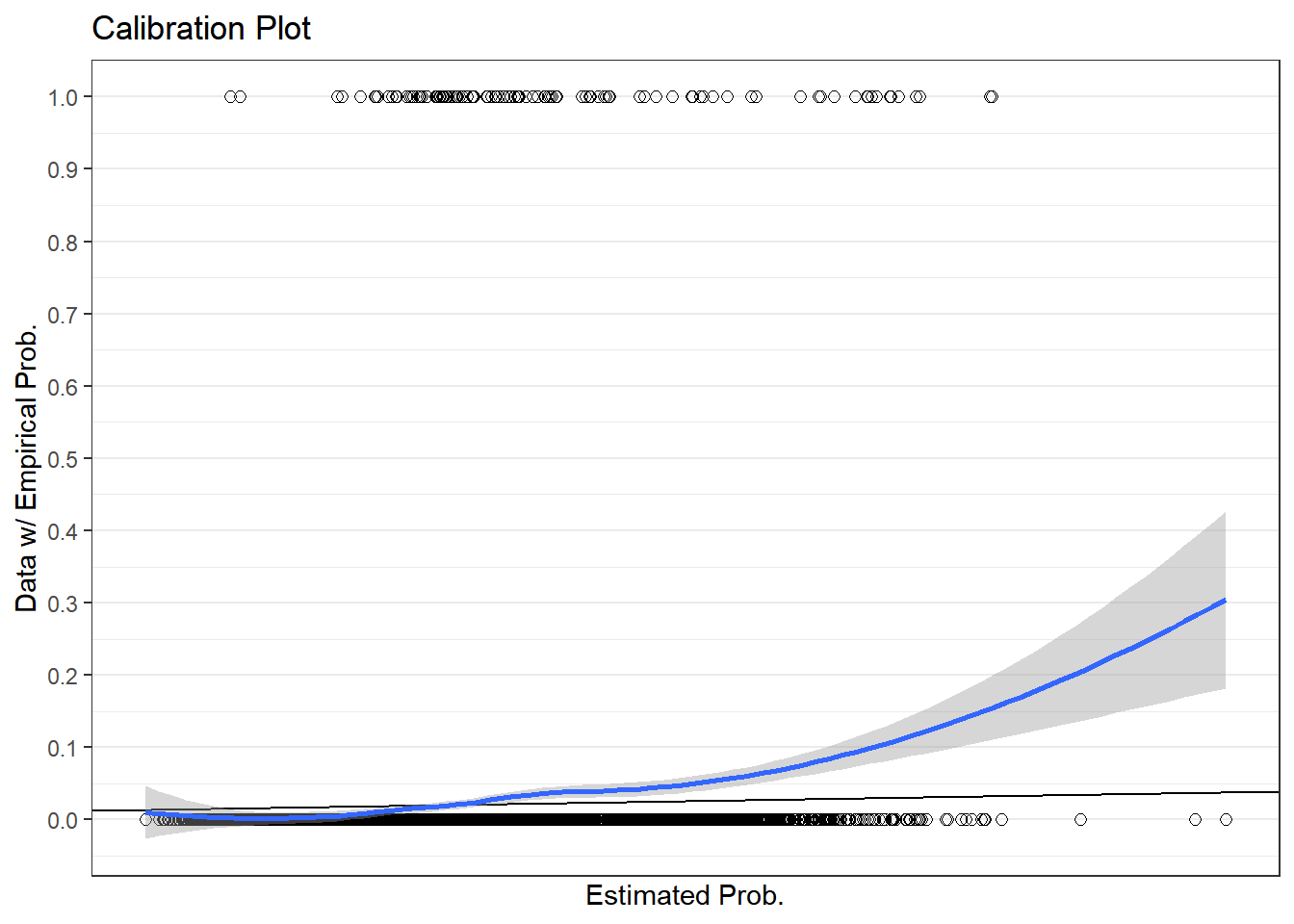


Supplementary Figure 8: calibration plot 1y stroke


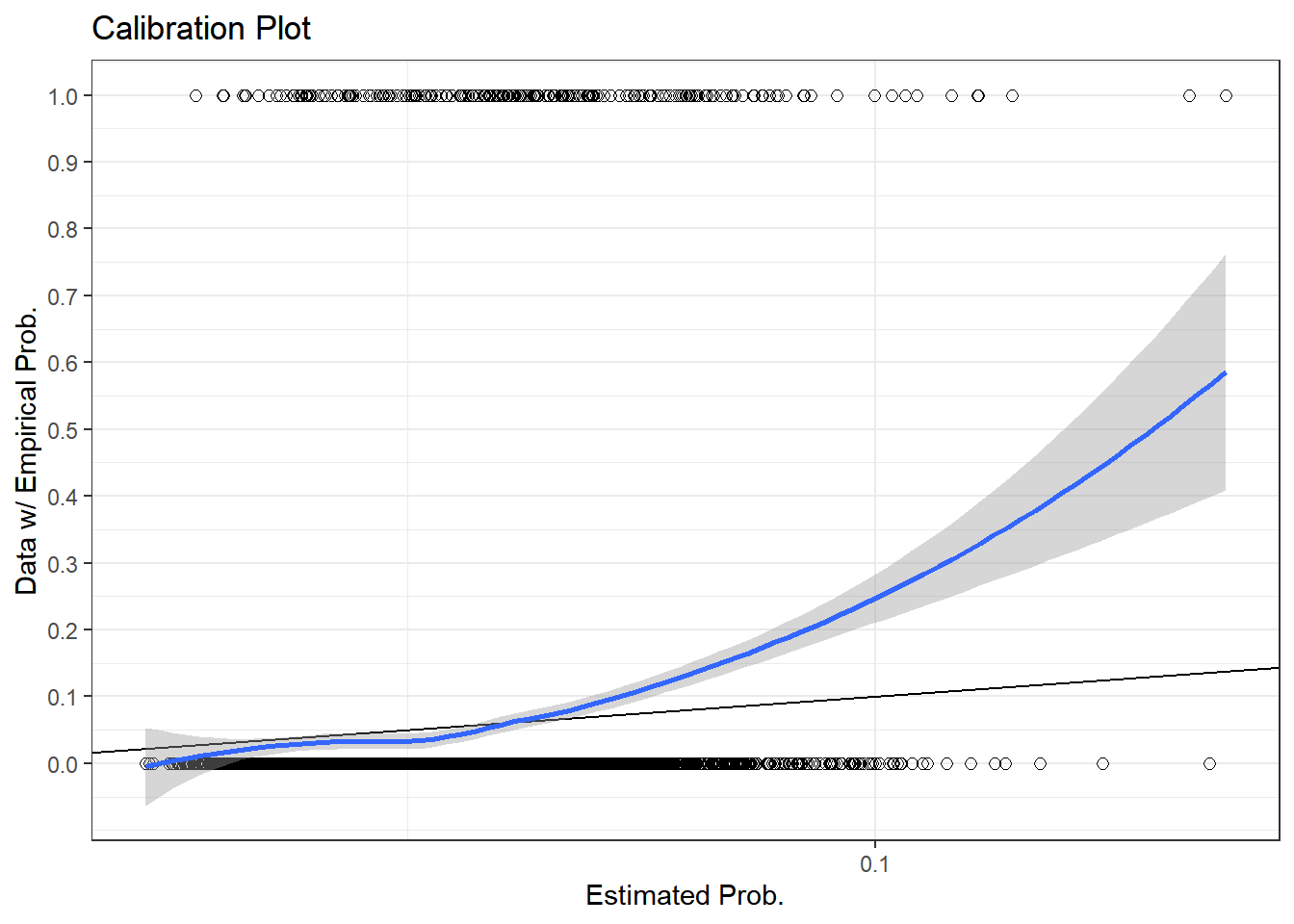

Supplement: ezaf016_Supplementary_Data [file ezaf016_supplementary_data.docx]
